# Supplementary material for: Are carbon emissions trading and green financial instruments synergistic? -Comprehensive quantitative research based on content analysis
Source: PLoS One. 2024 Mar 7;19(3):e0298601. doi: 10.1371/journal.pone.0298601 (PMC10919601; doi:10.1371/journal.pone.0298601)
Supplement: S1 Data — (ZIP) [file pone.0298601.s001.zip › Data/地级市Z_F工作报告.zip!/╔╜╬≈/┤≤═1⁄4╩╨/└┤╫╘╣1⁄2╓┌║┼í╛┬φ┐╦╩2╛▌═°í┐_2008.docx]

各位代表:

现在,我代表市人民政府向大会作工作报告,请予审议,并请市政协委员和其他列席人员提出意见。

一、过去五年工作回顾

五年来,在中共大同市委的正确领导下,在市人大、市政协的监督支持下,政府高举邓小平理论和“三个代表”重要思想伟大旗帜,认真贯彻党的十六大、十七大精神,深入贯彻落实科学发展观,全市经济社会等各个方面都取得了显著成就。

——经济发展持续增长。2007年,全市地区生产总值完成491亿元,年均增长3.2%。财政总收入和一般预算收入分别达到93亿元和36.7亿元,年均干净利落5.9%和26.6%。规模以上工业企业利税总额73亿元,年均增长22.5%。消费品零售总额185亿元,年均增长13%。五年累计完成固定资产投资718亿元,年均增长29.4%。经济增长的质量和效益不断提高。

——结构调整稳步推进。全市共完成重点工业调产项目83项,投资181亿元。煤炭行业关小扶大初见成效。地方煤矿由496座压减到270座,洗选能力由10%提高到60%,资源回收率由35%提高到65%。发电装机容量356万千瓦,增长1.5倍。冶金、化工、机械、建材产业继续发展。第三产业稳步增长,占到生产总值的42%。万元生产总值能耗、主要污染物排放问题明显下降。

——农业和农村经济健康发展。实施雁门关生态畜牧区建设,推进“两区”开发项目,夏进乳业、华晟果蔬、绿苑饮品等一批龙头企业初露头角。国家京津风沙源治理、首都水资源可持续利用、退耕还林和省六大造林工程累计完成生态建设任务515万亩,完成高速公路两侧绿化100公里,出省口景点绿化3402亩。农业综合生产能力逐步提高,发展节水农业56万亩,改造中低产田75万亩。新农村建设逐步推进。完成沼气建设2万户,解决61万人的饮水困难和安全问题,累计转移农村劳动力30万人。

——改革开放进一步深化。同煤集团全面重组,山橡公司并入蓝星集团,大同糖厂破产并购,大同化纤厂破产重组,大同煤业,大秦铁路A股成功上市。国省属大中型企业主辅分离。非公有制经济加快发展。外贸进出口总额完成2.7亿美元,年均增长40.7%。参加了沪洽会、港洽会、煤博会等大型招商活动,对外联系合作进一步扩大。

——城乡基础设施建设明显改善。五年累计投资9.8亿元,新建改造迎宾大道、大庆路、振华南北街等25条主次干道,改善小街小巷117条。实施煤矿棚户区改造和采煤沉陷区治理工程,一期已安置煤矿职工2.1万户。启动城市居民棚户区改造工程。公路建设实现历史性突破,得大高速、绕城高速公路竣工通车,县际公路改造630公里,市县公路基本实现二级化,村村通水泥路近一万公里,农村基本实现村通水泥路。大同机场建成通航。御河生态园建设成效明显,城市绿化覆盖率达到31.6%,人均公共绿地面积达到5.2平方米。主城区集中供热面积达到1400万平方米,二级以上良好天气达293天,退出了全国污染最严重城市行列。

——人民群众生活水平继续提高。2007年城镇居民人均可支配收入11508元,年均增长13.3%;农民人均纯收入3033元,年均增长10.4%,全市累计新增就业岗位28万个,城镇登记失业率低于4%,下岗职工累计实现再就业13.4万人,政府购买公益性岗位安置1.1万人。社会保障体系不断完善,城乡低保制度建立,农村“五保户”实现应保尽保。农村贫困和低收入人口由61.5万人减少到26.9万人,2.2万贫困人口实施移民搬迁。

——社会事业全面发展。科技进步对经济增长的贡献率提高。教育事业“两免一补”政策全面落实,“普九”攻坚任务圆满完成,新建、改建中小学校1458所,大同二中高中部建成,大同大学加快发展。文化事业繁荣活跃,耍孩儿、罗罗腔、恒山道乐、晋北鼓吹、广灵剪纸入选国家非物质文化遗产名录。卫生工作进一步加强,基本完成了101个公共卫生体系建设项目,新型农村合作医疗提前一年实现了全覆盖。竞技体育水平提高,群众体育蓬勃开展。人口自然增长率控制在8%以内。新增国家级重点文物保护单位9处。安全生产形势基本稳定。车防教育、优扶、双拥工作和民兵预备役建设卓有成效,信访、新闻、出版、民族、宗教、档案、史志、人防、老龄、残疾人、红十字会等各项事业都取得了新的成绩。

——民主法制建设进一步加强。坚持定期向市人大常委会报告工作,认真办理人大代表建议和政协委员提案,加强与各民主党派和群团组织的联系,广泛听取各方面的意见,科学化、民主化决策机制不断完善。加强普法和政府立法工作,五年来市政府共制定政府规章15件、修订4件、废止6件,提请市人大制定地方性法规(草案)17件、修订5件、废止6件。积极推进“平安大同”建设,治安状况明显好转。认真落实党风廉政建设责任制,坚持从源头上预防和制止腐败。加强审计监督工作,加大打击偷逃税费工作力度。

各位代表,过去的五年是我市改革开放和经济建设取得重大进展的五年,是我市经济实力进一步提升和人民群众得到更多实惠的五年,是全市上下解放思想、与时俱进、重振雄风、奋力赶超的五年。五年的不懈努力,创造了改革发展的丰硕成果;五年的生动实践,积累了继往开来的宝贵经验。五年的发展成就,是省委、省政府的市委正确领导的结果,是全市人民团结奋斗、扎实苦干的结果,是社会各界关心、支持、帮助的结果。

在此,我代表市人民政府向全市人民,向驻同解放军、武警官兵和公安干警,向各民主党派、人民团体,向所有关心支持参与大同建设发展的驻同单位和海内外各界朋友,表示衷心的感谢和崇高的敬意!

二、今后五年奋斗目标和2008年主要任务

今后五年,是大同实现科学发展、全面建设小康社会的重要时期。党的十七大对当前国际国内形势作出科学判断,对我国改革开放和社会主义现代化建设作出战略部署。我们必须部在更高的历史起点上,以更加宽广的国际视野,更加科学的战略思维,更加宏大的气魄胆识,适应国内外形势的新变化,顺应大同人民过上更好生活的新期待,把握经济社会发展趋势和规律,把大同未来发展放在经济全球化的大趋势下,放在全国、全省发展的大格局中,放在参与环渤海经济圈竞争发展战略定位上思考和谋划。

今后五年,大同面临总体有利的发展环境。当今世界正在发生广泛而深刻的变化,经济全球化深入发展,科技革命加速推进,和平与发展的时代潮流不可阻挡。当代中国正在发生广泛而深刻的变化,工业化、城镇化、市场化、国际化快速推进,经济体制深刻变革,社会结构深刻变动,利益结构深刻调整,思想观念深刻变化。当前山西正在发生广泛而深刻的变化,“四条路子”、“三个跨越”、“四大攻坚”的大战略深入贯彻,国内外能源需求大市场带来的空间和机遇,又好又快发展成为全省上下的共识。面向未来,大同有改革开放以来奠定的坚实基础,还有煤炭工业可持续发展试点、振兴东北老工业基地、西部大开发比照政策等重大机遇。

大同人民宽容、敦厚、勤劳、善良的优秀品质,人心思变,人心思上,人心思干,人心思富的大势,民心可用,民意可依,民气可成。同时,我们也必须清醒地看到,我市经济社会发展还面临一些突出矛盾和问题,支柱产业中新型产业比重过小,多种所有制经济中民营经济比重过小,区域经济结构中非煤产业比重过小,经济发展方式中集约型增长比重过小,经济发展总量中县域经济比重过小,对外开放格局中大企业、大集团引进比重是过小;生态环境问题过多,民生社会事业欠帐过多,国有企业改革发展困难过多,城市基础设施建设滞后过多。概括起来就是“六个过小、四个过多”。

这些情况表明,在现代新大同建设的宏伟征程中,机遇前所未有,挑战前所未有。必须进一步增强机遇意识、责任意识、忧患意识,跳出大同看大同,立足山西看大同,放眼全国看大同,融入世界看大同,勇于用新的信念、理念、观念指导科学发展,善于用新的思想、思维、思路把握后发优势,敢于用改革、开放、创新的大举措谋求超常突破,奋力开拓大同更为广阔的发展前景。

今后五年政府工作的总体要求是:高举中国特色社会主义伟大旗帜,以邓小平理论和“三个代表”重要思想为指导,深入贯彻落实科学发展观,紧紧围绕“转型发展、绿色崛起”这一主题,强力推进工业化转型、城市化提升、生态化崛起、国际化拓展、科学化发展,加快建设全国重要的新型能源基地、先进制造业基地、现代服务业基地,精心打造世界文化遗产旅游城市、国家历史文化名城、国家风景名胜区三大品牌,在山西崛起中争先进位,在中部崛起中追赶前进,努力建设经济景气指数高、历史文化特色浓、休闲宜居景观美、投资创业环境好、社会和谐人气旺的现代化区域中心城市。

未来五年的主要奋斗目标是,到2012年,地区生产总值突破1000亿元,年均增长15.3%;财政总收入达到300亿元,年均增长26.4%;城乡居民收入实现翻番,年均增长14.9%,分别达到23016元和6066元;人口自然增长率控制在6.5%以内,空气质量达到国家二标准。

实现今后五年经济社会发展目标,2008年要着力做好以下工作。

(一)以资源型城市转型为着力点,推进工业强市发展战略

大同是个传统工业城市,振兴大同必须振兴工业。工业化是现代化的核心、城市化的内涵、社会文明化的灵魂。要把传统工业新型化、新兴工业规模化作为大同崛起的关键,强力推进工业园区建设、工业项目引进、工业问题扩张、工业结构调整、工业产权整合、工业产业升级、工业产品升位、工业品牌打造、工业人才培养、工业市场开拓的攻坚战。

推进煤炭、电力产业新型化,巩固和发展国家重要的综合能源基地。更加重视和充分发挥同煤集团龙头老大的带动力、集聚力、辐射力,强化国家能源基地的产业支撑力、政治影响力。要创造最宽松的环境、最优惠的政策,支持同煤集团进一步做大做强。到2012年,同煤塔山、同忻、马道头、东周窑四个千万吨以上矿井全部建成投产,煤炭产销量达到1.6亿吨,煤炭产量达到1.2亿吨,其中大同地区矿井产量1亿吨,销售收入达到600亿元,继续保持全国第二大煤炭企业的地位。支持同煤集团多元化、综合性拓展,电力、化工、冶金、机械、建材、铁路运输多业并举,向特大型综合能源集团迈进。依托同煤等大型企业集团,整合左云、南郊等地的小型矿井,并小,提高煤炭机械化装备水平,建设现代化矿井,建立煤炭安全生产的长效机制。

年产量不达30万吨的煤矿逐步关闭,确保地方煤炭产量稳定在5000万吨左右。支持国电电力、大唐电力、国投、山西国际电力、格盟国际电力加快发展,形成煤电一体化并向下游延伸的产业链。国电电力要加快三期、四期工程建设,到2012年建设成为总装机容量500万千瓦大型火电基地,全市总装机容量力争突破1000万千瓦。煤电一体、装备先进、环保节能、多元发展,让中国“煤都”的品牌在新型工业化的历史条件下重放异彩。

大力推进自主创新,强化装备制造业基地建设。自主创新是转变发展方式的中心环节,装备制造业是大同新型工业化的希望所在。支持北车集团大同公司、大齿、山柴等龙头企业,加强国际科技合作,增强自我研发,自主创新的能力。同车公司与法国阿尔斯通公司合作开发的八轴大功率交流传动电力机车,代表了当代世界一流水平,到2012年销售收入将突破100亿元,成为国内轨道交通装备的核心制造商之一和山西省轨道交通装备产业化基地,产品研发能力、生产制造能力、质量控制能力、企业获利能力全面接轨国际水平。

建造以大齿集团为龙头的大同汽车零部件产业集群,大力推动大齿集团与东风公司战略重组,组成资本联结、优势互补、资源共享、产品依赖、研发超前的紧密型合作,打造具有国际竞争力的汽车零部件生产基地,带动大同汽车改装等产业集群发展。积极寻找大同装备制造业的发展契机,推进与中国重汽集团等国家特大型企业的合作。结合“863”重型商用车变速器开发项目,不断提升自主创新能力,升级为国家级支持中心。到2012年,大齿变速箱销量达到25万台,年销售收入突破30亿元。

推动山柴由军品主导向军民协调发展转型,加强与德国MTU公司的国际合作,重点抓好“高技术、新能源、大功率”为特色的军民通用型产品,着力培育以煤层气为主要燃料的节能环保型发电机产品,寻求与大集团、大公司配套合作,形成市场批量生产能力,带动地方经济发展。培养和引进一批核心竞争力强、拥有自主知识产权的制造业集团,是资源型城市转型落地的根本。

强力实施“同药战略”,培植医药工业的支柱地位。医药工业是国际公认的朝阳产业,具有成长速度快、成熟期短、机遇峰值周期长、市场前景广阔的特点。大同气候干燥凉爽、原材料丰富、产业基础较好,发展医药工业有独特的优势。要抓住世界医药产业重组的专利药保护到期的机会,研究制定同药发展战略,打造华北“药都”。优化原料药、成品药、中药等产业的空间布局,形成将有共享,配套互补、环保达标、循环利用的产业集群。制定扶持鼓励医药工业发展的政策措施,在土地、信贷、税收、基地建设、上市融资等方面予以倾斜,实施封闭管理。建立医药产业发展基金,支持新药研发、品牌创优、市场开发等风险投资,扶植威奇达、普德等龙头企业快速发展,吸引国际资本、省外资本进入大同,加快企业集团化、产业规模化、品牌国际化进程。建立权威高效的组织领导机构,设立同药产业办,形成统一领导、协调高效、推动有力的工作机制。力争2012年医药工业销售收入突破100亿元,做大做强,整体创新,成为大同的支柱产业。

加快传统装备业优化升级,推动经济发展方式转变。坚持工业化与信息化融合,以信息化带动工业化。加快用高新技术和先进适用技术改造提升冶金、建材、化工等传统产业。推进同煤钢铁公司与首钢的合作,力争2010年产量突破300万吨,销售收入突破100亿元。推进大同水泥集团破产重组,引进名列全国建材行业前茅的冀东水泥集团,两年内建成环保达标、技术先进、产品优良、年产500万吨的水泥生产线。推进山橡公司氯丁橡胶生产线搬迁改造,引进国外先进技术,清洁生产,减少污染,循环发展,扩大规模,2012年氯丁橡胶总产达到9万吨,销售收入达到20亿元,建成具有国际先进水平和国际竞争力的龙头企业。

发挥园区载体功能,促进产业集聚发展。园区是工业强市的主战场。以经济建设为中心,就是以园区经济为中心。要按照园区现代化、产业高新化、布局专业化、机制市场化的原则,加快实施园区突破工程。继续加大经济开发区开发的力度,扩区升位,加速发展,打造平台,重塑形象,真正成为大同经济快速增长的引擎,资源型城市转型的产业隆起带。抓好塔山工业园和阳高龙泉工业园,提高规划和开发水平,配套基础设施建设,把经济开发区和工业园区建成办事最快、效率最高、服务最好、条件最估、富有吸引力、集聚力的发展平台。鼓励各县区按照集中、集聚、集约的原则,创办工业园区,推动工业向园区集中,推进园区特色化发展。

二十多年前,大同以工业城市的辉煌,被国务院确定为全国13个较大城市之一,各闻天下。当历史的脚步走到今天,应当承认大同相对落后了。大同要富强,富强靠工业。我们必须以卧薪尝胆的决心,壮士断腕的勇气,励精图治的作风,与时俱进的精神,上下同心,合力攻坚,建设新型工业化历史条件下的工业强市,创造超越前人、无愧后人、无负时代的新篇章。

(二)以文化旅游产业崛起为战略重点,推进现代服务业建设

文化永远是一个城市赖以生存与发展的核心竞争力。丰富的历史文化遗存,是大同不可再生、不可替代的第一珍贵资源。强力推进以中国历史文化名城为中心,以世界文化遗产云冈石窟、国家风景名胜区北岳恒山为两翼,以浑源永安寺、汤头温泉,新荣得胜堡、方山永固陵,大同火山群、土林,阳高许家窑遗址、大泉山、云林寺,广灵水神堂,灵丘曲回寺石像冢、平型关遗址、觉山寺,天镇慈云寺、新平堡、古长城,左云古城墓群为群体的文化旅游大产业,是实现富民强市的重中之重,是建设现代服务业的第一要务。文化旅游产业是一个包容性、运动性、多元性和辐射性非常大的产业。旅游整合世界,人类共享文明。文化旅游产业可以兴大同、富大同、名大同,让世界了解大同,让大同走向世界。

加快历史文化名城保护和修复的步伐。历史文化名城是一个鲜活的、有生命力的整体,真实性和完整性是其生命力的基本特征。大同古城是个水平城市,在大片低矮的四合院民居中间有节奏地矗立着钟楼、鼓楼、魁星楼、太平楼以及各个城门楼、角楼等标志性建筑,城市空间平缓开阔,天际轮廓错落有致,构成古城独特的艺术景观。真实性就是保留历史的原貌,整体性就是保护好历史城区的整体环境。名城保护要在规划的指导下,按照文化旅游大产业的功能要求,科学、高效、有序推进。今明两年,首先修复上下华严寺、善化寺、文庙、关帝庙、清真寺、法华寺、帝君庙、纯阳宫、玄真观、东城墙、城楼、角楼、瓮城、鼓楼东街商业步行街、东南隅部分民居,初步形成名城东部轮廓。2010年完成总镇署、府衙、代王府、南城墙整体、东小城、部分商业街改造包装。到2012年完成西城墙、北城墙整体修复,拆除、改造不协调建筑,恢复名城历史风貌和城市肌理,形成集展示、浏览、体验、娱乐、教育、休闲、购物、居住、饮食于一体的文化旅游大产业,基本完成名城保护和修复的任务。

全方位精心打造世界文化遗产云冈石窟旅游大景区。云冈石窟是大同的生命之根、文脉之本、风貌之基、无价之宝、不朽之魂。要按照世界眼光、国际水准、大同特色的要求,搞好大规划,建设大景区,打造大产业。大景区范围的村庄全部搬迁,不协调建筑全部拆除,进行大规模、高水平的绿化、文化、美化,建设与世界遗产相匹配、国际一流的大景区环境。丰富景区的文化内涵,完善景区的服务功能,提升景区的核心价值,配套北魏文化博物馆、吃住行游购娱等服务功能,充分展现云冈雄伟壮阔的旷野之美,吞吐万汇的深远度量,撼人心肺的文化张力。

全面提升国家风景名胜区北岳恒山的景观水平。恒山是“五岳”名山之一,是人与自然精神联系的圣地,是江山社稷的代表,具有传世性的科学、美学和历史文化价值。悬空寺是恒山的灵魂,鬼斧神工,骇人心目,“此物只应天上有”,是人类遗产的奇迹。要按照“区内景、区外景”的规划原则,精心整治和打造景区内外环境,拆除有碍观瞻的不协调建设,搬迁影响景区环境的村庄及各种服务设施,按照“整旧如旧”的原则,全面系统地改造提升山水寺庙建筑,景区整治外扩到神溪湿地、永安寺、文庙、圆觉寺、栗毓美墓和历史街区,改善景区道路环境,进一步提高国家风景名胜区的自然度、美感度、灵感度,还锦绣河山之完美形象,复兴中华山水文化精神。

统筹发展文化娱乐、商贸流通、宾馆餐饮、现代物流等各类服务业。继承和发展剪纸、铜器、泥人、绢人、皮影等大同特色的民间艺术,丰富广灵剪纸博物馆,不断研究和创新艺术产品,弘扬和扩大民间艺术的影响力。创办广灵剪纸等民间艺术技校,下大力培养民间艺术传人,让大同民间艺术代代相传。加强对两汉、北魏、辽金、明清以及魏碑、平城遗址等历史学、建筑学、考古学的研究,繁荣学术讨论和文化艺术创作。发掘、整理、提高耍孩儿、罗罗腔等非物质文化遗址,发展与文化旅游产业相适应的优秀地方传统剧目。强化报纸、电视、网络等新闻媒体的宣传营销,不断提升和扩大大同文化的软实力。整合资源,做大做强以云中商场为龙头的商贸流通中心,强化晋、冀、蒙区域辐射,构筑以大同为中心的北部城市群物流核心圈。发挥“吃在大同”的传统餐饮优势,大力发展具有大同地方特色的酒店业、餐饮业,为“游在大同”增添舒适性、快乐游条件。按照全省以太原为中心,以大同、侯马为主要节点的物流布局,充分发挥大秦铁路股份公司的龙头作用,加快“运输组织现代化、技术装备现代化、运营管理现代化”进程,今年营业收入突破225亿元,两年内实现年运量突破4亿吨,构建现代煤炭物流体系。推进标准化城郊农贸批发市场建设,完善城区商贸配套服务功能。努力推进“万村千乡市场工程”,新建村级店200个,完善农家店配送中心建设。

大力发展现代金融业。现代金融业是现代服务业的中心内容。市场经济时代是一个资本化时代。资本市场既是现代市场经济发展的必要条件,也是现代市场经济发达程度的重要标志之一。大同未来的可持续发展,离不开一个强大的资本市场。深化金融体制改革,推进大同商行资本结构、投融资结构和公司治理结构的完善,充分发挥工、农、中、建等四大商业银行和农村信用社的作用。把发展资本市场作为一项重要战略任务,推进股票、债券、商品期货和金融衍生品市场的全面发展,培养大批了解国际动作、服务于大同经济需求的专业人才,不断扩大直接融资为主的金融体系构建,让更多的企业通过资本市场充分发现和提升自身价值,更好地融入到国际经济循环中去,为企业做大做强提供广阔的平台。让更多的群众拥有财产性收入,分享经济增长的成果。

历史文化遗产是大同昨天的辉煌,也是今天的财富,更是明天的希望。大同文化旅游产业振兴之时,也是民族传统文化复兴之日。这是继北魏举世无匹的文化创造之后的又一个文化盛典。未来五年,大同文化旅游产业要做到全省最强;未来十年,大同文化旅游产业要成为继煤炭之后的又一个支柱产业。

(三)以特色化魅力城市为目标,推进城市现代化建设

城市是人类文明进步的产物,是先进生产力的载体,是区域竞争的龙头。市场经济是城市化经济,也是龙头带动型经济。市场竞争集中体现在城市竞争。大同作为全省第二大城市和历史文化名城、全国较大城市,必须站在时代的制高点上,以宏大的国际视野制定和完善总体规划,按照“一轴双城,分开发展;古今兼顾,新旧两利;传承文脉,创造特色”的要求,大力推进名城保护、新区建设、旧城改造、矿区提升,提高城市整体水平。

尽快启动御东新区建设,强化全省第二大城市的地位。御东新区是大同二次创业的新起点,要按照前瞻性、生态性、文化性、独特性的要求,高起点规划、高标准建设、高强度投入、高效能管理,把新区建设成为基础设施先进、生态环境优美、新型产业集聚、创业居住适宜的现代化新城。新区开发要统筹规划、分期开发、基础先行、滚动发展。今年力争完成总规修编的控制性详规,明年完成御东大道、兴云路、南三环和操场城东街延伸段等10条骨干道路建设,完成二中、实验中学扩建工程,启动商贸中心、金融中心、住宅中心等工程建设。2010年完成南三环桥、操场城桥和兴云桥改造等骨干桥梁,以及水电气暖等主要基础设施建设,完成第五人民医院和十六中新建工程,完成文瀛湖景观、御河生态园景观建设,实现行政中心东移,完成部分商贸中心、金融中心和住宅中心建设,新区城市框架。2011年到2012年,开发重点向全面建设延伸。大剧院、会展中心、体育中心、图书馆、文化教育中心等第三产业初具规模,新型工业和高新技术产业有较大发展,大同机场基础设施和客运能力全面提升,临空产业启动发展,初步形成新区格局。

继续加大旧城改造力度,提升老城区基础设施建设水平。今年在完成御河南北路改造延伸、迎宾东西路、宾西路改造延伸、向阳东西街改造延伸、西三环改造拓宽、南三环改造、西三环安置小区道路配套、同左路改线及云冈峪景观改造等重点工程的同时,超前谋划、规划、设计,更充分地做好其他道路改造的前期准备。明年抓好新建南北路、新开南北路、同丰路、工农路、北环路、新胜东西街、操场城东西街以及小街小巷的高标准政治改造。后年全面完成旧城区道路等基础设施改造建设,实现三年基本完成旧城区改造的既定目标。

加大矿区改造提升的力度。充分发挥同煤集团的建设力量,完善道路、电力、通信、水暖、供气等基础设施建设,提升城市功能,改善生态环境,强化区域服务中心职能,大力推进塔山工业园新型工业发展,富集要素,集聚人气,力争三、五年内建设成为新的城市副中心。

城市建设必须坚持规划引导和控制的原则,树立和强化规划的权威性、原则性和严肃性。规划是建设的龙头,是一个城市不败的基础。规划决定城市。大同城市建设首先不能输在规划上。规划就是财富,文化就是灵魂,历史就是永恒,生态就是生命,人本就是根本,协调就是美德。我们在城市规划和建设上既有成功的经验,也有深刻的教训。概括起来就是一句话,高水平就是财富,低水平就是包袱。大同在大规模的城市建设中失败不起,只能创造财富,创造先进水平。规划是政府第一资源,第一责任。掌握规划、建设、管理权利的领导者、工作者,必须以多留遗产、少留遗憾的历史眼光,宁挨一时骂、不挨千秋骂的责任担当,不求做大官、但求做大事的理想情怀,严格按照规划大于权利,逻辑大于存在,历史大于现实,生态大于经济,整体大于局部,长远大于眼前,公共大于个体的原则,把好规划的审批关、执行关、落实关,引导和规范开发商实现开发和城市共赢互利,为社会创造财富,为人民创造幸福,为人生创造价值。

土地是财富之母,也是经营城市、建设城市的核心要素。搞好城市建设,必须严格规范土地市场。要加强土地资源储备管理,坚持一个龙头进水,垄断土地一级市场,农村集体土地进入市场,必须由政府按规划限价征用储备。开发商取得土地使用权,必须通过政府土地交易中心招、拍、挂,在公平、公正、公开的市场竞争中合法取得,一个闸门放水。任何农村组织、企业、单位与开发商私自进行的土地交易,均属违法行为,必须坚决制止和纠正。土地交易的腐败是最大的腐败,土地收益的流失是公共利益最大的流失。要坚持规划引导土地开发的原则,加强土地一级开发,变生地为熟地,实现土地收益最大化,不断积累城市建设的物质基础,让全市人民共享土地升值、增值的成果。

城市建设“三分建七分管”。要按照“精心、精细、精品”的要求,坚持不懈地加强城市管理工作。市容市貌就是城市品牌、文明形象。要继续深入开展城市环境综合整治,强势发动、强力推进、强制规范、强行入轨,做到全面覆盖、长效管理、不留死角、永久保洁。违法建设是现代新大同的大敌,是城市建设的破坏性力量,必须以坚强的决心、对历史负责的态度、铁的手腕予以控制和纠正。要以改革的精神,合理调整行政区划,进一步理顺城市规划、建设、管理体制,整合规范权利配置,理顺关系,优化职能,提高效能,形成权责一致、分工合理、决策科学、执行顺畅、监督有力的城市管理体制。要按照“两级政府、三级管理、权利下放、重心下移”的原则,充分发挥区级政府管理城市的职能。加强街道社区建设,强化社区在城市管理中的基础作用。

在全球化城市发展的浪潮中,创造特色化魅力城市,彰显蓝天之下、大地之上的完美艺术形象,这是一项伟大而崇高的事业。需要有诗情与画意相融铸的艺术修养,自然情趣与文化精神相统一的审美眼光,尊重历史与忠诚事业相结合的献身激情。我们要按照特色化的追求,力争城市建设一年一个样,三年大变样,五年变新样。

(四)以城市统筹为支撑点,推进农业和农村工作

“三农”问题直接涉及到我市50%人口的利益,始终是我们工作的重中之重。“三农”问题的实质是农民增收,重点是城乡统筹,关键是转移农民,抓手是农业产业化、现代化。

突出“三优”重点,提高农业现代化水平。围绕优势产业、优势基地、优势产品,在确保粮食安全、粮食产量稳定70万吨左右的基础上,强力推进畜牧、果蔬、优质杂粮、特色农产品四大主导产业,发展特色高效农业,提高农业规模化、集约化、专业化、产业化水平。按照“一个产业、一个规划、一套政策、一批龙头、一群农户、一片基地”的要求,落实农业产业化大发展的动力机制和组织体系。以夏进乳业、百草香肉业等12户畜产品加工龙头企业,带动56个标准化奶牛养殖园区、100个养羊专业村、5万头和50万只以上规模养猪、养鸡乡镇。以绿苑、华晟等7户果蔬加工龙头企业,带动阳高、天镇、大同、浑源等22万亩杏树基地,南郊、阳高、天镇、广灵等30万亩蔬菜基地。以佳鑫、东方物华等15户杂粮加工龙头企业,带动30万亩谷类、50万亩马铃薯、20万亩荞麦、5万亩苦荞、50万亩豆类优质杂粮基地。以万生黄氏、大同黄花、大同茂源天然色素、广灵北野菌业等8户特色农产品加工龙头企业,带动28万亩黄氏、5万亩黄花、2万亩万寿菊、1万亩食用菌特色农产品基地。形成抓龙头上规模,抓特色建基地,抓服务建体系,抓典型带全面的产业化机制。

壮大县域经济规模,强化小康社会建设的基础工程。县域经济是国民经济的基本单元,体系完整、职能完备、决策优势、分类指导、梯次推进的原则,积极探索和创新县域经济社会发展机制。在县域空间开发形式上,要由“群马拉车”转向“大马拉车”,加快实施县城建设开发提升工程,重点把县城规划做好,规模做大,实力做强,功能做全,产业做优,环境做美,形成以城带乡、带村的发展大格局;在县域经济增长动力上,要由依靠“官资”为主转向依靠“民资”为主,以启动民间资本为杠杆,以民间投资和消费为支点,撬动县域经济发展;在县域经济的增长方式上,要由主要依靠外延转向内涵、外延并重,更多地依靠科技进步,发展现代农业;在县域经济比较优势的开发上,要由单纯注重底下“黑色”资源转向地下地上资源开发并重,加大绿色无公害产品、历史文化旅游等“绿色”资源综合开发;在县域经济市场定位上,要由一味内向拓展转向内外结合、双向启动,并着力开拓外部大市场,充分发挥大市场对经济增长的引导力、牵引力和拉动力。大同11个县区中有5个是国家扶贫开发重点县,共有贫困人口26万余人。在县域经济发展中,要始终把脱贫致富作为重点,力争两年内每个扶贫开发县培植1—2个销售收入超亿元的龙头加工企业,变“输血式”扶贫为“造血式”扶贫。

加强农村基础设施建设,扎实推进新农村试点工作。基础设施建设是新农村建设的物质基础,也是农村亟待加强的薄弱环节。要认真落实中央“三个明显高于”的政策,保持财政支农政策的连续性和稳定性,完善财政支农资金稳定增长的长效机制。试点村要发挥示范效应,重点村要提高建设水平,配套建设社会事业“五个一”工程,开展道路通达、信息网络、科技培训、供销服务和文明新风“五进村”活动,努力改善农村生产生活条件。

农业是安定天下的基础产业。民以食为天,食以农为本。在市场经济条件下,农业作为一项弱质产业,虽然在国民经济的份额中逐渐降低,但这丝毫不能动摇“三农”的基础地位。要时刻牢记,我们在这个世界上可以创造很多辉煌,但永远不能忘记脚下的起点,不能忘记养育我们的土地和农民父老。

(五)以改革开放为动力点,激活招商引资的能动力量

加快大同富民强市的步伐,必须动员一切可以动员的力量,调动一切可以调动的资源,敞开引进外来先进思想的同化和渗透,引进外来人才的挑战和竞争,引进外来生产要素的聚集和活力,不断把改革开放提高到一个新水平。

全力实现招商引资新突破。发展是第一要务,招商引资是第一要事。政府各级领导干部要牢固树立“招商引资是第一政绩”的理念,实施招商引资“一把手”工程,明确“一把手”在招商引资中的“第一责任”,以“第一责任”形成“第一政绩”,以“第一政绩”衡量“第一能力”。解放思想是招商引资、对外开放的“头道工序”。要通过思想大解放,在全市上下形成一股冲击波,冲破养尊处优、懒惰疲沓、不思进取、看摊守业的思想意识,强化有大追求、干大事业、谋大发展的勇气和魄力;冲破“唯上”、“唯书”,怕失误而不怕丧失机遇、怕负责任而不怕发展慢的精神状态,倡导快人一拍掌握主动,先人一步赢得先机,敢为天下先,敢吃第一个螃蟹的拼搏精神,让思想之花迸发,让创新精神涌流,让创业热情激荡,让招商硕果累累。

招商引资要坚持依托北京发展大同的战略思想,在环渤海经济圈找机会、下重力。推进经济、科技、文化、教育、卫生等多层次、多领域的合作,把北京与大同长期形成的地缘优势、人文优势,转化为经济发展优势、科技进步优势、人才交流优势、资源共享优势。鼓励和选拔优秀干部投身招商引资第一线,瞄准大集团、大企业,蹲点招商、上门招商、跟踪招商、情感招商,确实提高招商引资的有效性、务实性和创造性。

强化企业在招商引资中的主体作用。充分发挥大同中小企业的能量,更高水平地“引进来”,更大步伐地“走出去”,在开放中加快民营经济发展,打造“为大同喝彩”的团队精神。要创造民营经济大发展的环境和政策,支持、鼓励和帮助民营经济做大做强。大同是中国的大同,更是大同人的大同。大同的发展离不开大同人的创造和智慧。政府热切希望那些有能力、有实力、在市场经济的大潮中走先一步、率先发展致富的大同人,那些大同杰出的优秀儿女,怀着热爱大同、发展大同、报效大同的满腔热情,回到家乡来,回到养育自己的土地上,投资创业,为建设美好大同贡献自己的力量。老板不在大小,能回大同就行;投资不分多少,钱花在大同就行。家乡是投资热土,家乡能成就辉煌。谋利应谋大同利,求名当求万世名。

着力打造招商投资的大环境。市场经济就是环境经济,招商引资的优势就是环境优势。当今区域竞争已经从比区位、比资源向比环境、比服务转变。要在政府机关和全市上下形成这样一种共识,不抓软环境就是失职,抓不好软环境就是不称职;谁损坏大同的形象,谁破坏大同的软环境,谁就是和全市人民过不去。要下大力、出狠招,建立严厉的问责制度、严格的限时办结制度、严肃的服务承诺制度,努力将大同打造成创业最宽松、最快捷、社会最文明、服务最优良,低交易成本、低生产成本、低行政成本、低社会成本的“四最四低”地区,吸引更多的投资落地大同,开花结果。以奥运圣火传递大同为契机,大力弘扬“更高、更快、更强”的奥林匹克精神,推进全社会精神文明建设,树立开放大同、文明大同的时代风貌。

历史的经验告诉我们,改革开放是决定当代大同命运的根本所在,招商引资是加快发展、奋力崛起的必由之路。只有解放思想,才能与时俱进;只有改革开放,才能富民强市;只有招商引资,才能成就伟业。

(六)以绿色崛起为基本点,推进生态文明建设

大同落实科学发展观,第一要义是发展,核心是以人为本,基本要求是创造环境友好型的生态文明新大同。“生态城市,我们的家园”,是大同经济社会发展的核心价值观和神圣法则。全面落实环保优先的方针,从根本上转变经济发展方式,实现由“环境换取增长”向“环境优化增长”转变,做到经济建设与生态建设一起推进,产业竞争力与环境竞争力一起提升,经济效益与环境效益一起考核,物质文明与生态文明一起发展。

落实环境保护的工程措施。加大城市基础设施建设的力度,明年要坚决落实市区集中供热百分之百覆盖,垃圾、污水百分之百处理,民用天然气百分之百使用。下大力治理册田水库、文瀛湖、御河、十里河,截污治污,净化水质,环湖环河绿化,恢复生态系统,三年实现碧水青山目标。

抓好污染企业治理。落实节能减排各项措施,坚决淘汰落后生产能力,限期治理、搬迁污染企业。主城区的工业企业要逐步搬迁,“退二进三”。经济开发区医药原料企业要加强“三废”达标处理,不达标的必须实施异地搬迁,确保御东新区生态园林城市的“净土”品质不受损害。长期对大同城市水资源和空气造成重大危害的企业,必须限期实施搬迁。133家重点排污企业要按照省政府的要求,在今年年底前必须实现达标排放,否则一律关闭。

大力开展绿化造林活动。绿化是城市第一形象,生态是城市第一基础设施。要树立“不求最大,但求最佳”,“不比形态,但比生态”,“不比高楼大厦,但比绿化美化”的理念,大规模、高水准推进绿化、美化。道路绿化要做到见路栽树、见土植绿,因路造景、因景配绿,移植大树、立见成效。道路拓宽改造原则上做到只拆不建,增加绿地空间,建设绿地广场。抓好环城高速沿线、铁路沿线、国省干道沿线绿化工作,造绿添景,连片、大片植树造林,荫及于民。开展庭院绿化创建工作,推进绿化美化向社区、小区、单位、院落和家庭延伸,见缝插绿,土不露天,连点成线,扩线覆面。要推广驻同部队植树造林、绿化管护的经验和标准,让森林走进城市,让城市融入森林,让大同迈向自然和谐的生态文明。

生态文明是以人为本的理性自觉,科学发展观的核心内涵,当今时代的普世价值观。人是最宝贵的,人生存与发展依赖的生态环境是最宝贵的。我们先要绿水青山,才能要金山银山。

(七)以改善民生为根本点,推进社会各项事业建设

发展之道,为政之要在安民、富民。“天下顺治在民富,天下和静在民乐”。要牢固确立以人为本、以民为主的理念,致力于让人民群众成为经济发展的主体、民主政治的主人、文化创造的主角、社会进步的主力,下大力解决好民生的迫切问题,使全市人民成为现代新大同建设的最大受益者,真正做到发展为了人民、发展依靠人民、发展成果由人民共享。

随着名城保护、旧城改造、新区建设的深入推进,被拆迁群众的住宅安置问题,成为当前和今后一个时期最重大、最紧迫的民生工程。实现好、维护好、解决好人民群众的切身利益,是政府一切工作的出发点、落脚点和根本点。要将拆迁群众的合理安置和居住品质提升,贯穿于城市改造拆迁的全过程,真正做到拆迁谁、改变谁、造福谁,让拆迁给人民群众带来新生活的机会,坚持城市建设与善待百姓的统一,共享城市建设的成果。对符合廉租住房条件的特困户,要给予住房保障。为了体现以人为本、和谐拆迁、惠及百姓、造福人民的指导思想,政府已经出台了城市住宅拆迁安置的若干规定,具体办法是:零成本以旧换新,补贴价保障住房,成本价以小换大,限制价让利于民,市场价求大求好。安置新区的建设标准是:基础设施配套、城市功能完善、生态环境良好、居住品质提升。政府公开向社会承诺,请人民群众监督。

要大力发展教育、卫生、科技、体育等各项社会事业,解决好上学难、看病难、就业难等问题。优化教育布局,扩大教育规模,提高教育质量。今明两年要完成一中、五中、六中的新建和二中、实验中学、北岳中学的改扩建。拆迁安置小区要优先保证教育配套。今后凡开发面积超过10万平方米的住宅小区必须配建幼儿园和小学,做到“新帐不欠,多还旧帐”,实现人口增长与教育需求的平衡。全面落实城乡“两免一补”政策。继续支持大同大学建设,强化城市名片的品牌效应,积极筹建大同师范高等职业教育学院。整合发展医疗卫生事业,推进名医名院对闲置医疗资源的整合重组,走医疗事业集团化的路子,做大做强做优医疗卫生事业。加快社区医疗网点建设,提供便民、利民医疗服务。加强科技创新和普及工作,促进科技成果转化。积极发展体育事业,加快建设现代化的御东新区体育中心。加强人口和计划生育管理,稳定低生育水平。进一步做好民兵预备役和“双拥”工作。

加快社会保障体系建设和再就业工程建设。严格规范国有企业改制和破产工作,坚决防止和纠正国有资产流失。逐步解决好破产、停产企业职工养老和医疗保险问题,真正落实老有所养、病有所医,推动建设和谐社会。实施积极的就业政策,推进创业就业工程。积极倡导全民创业,以创业带就业,拓宽就业渠道,激发全社会创造活力,使大同成为“百姓创家业,能人创企业,干部创事业”的一片热土。

人民群众是我们的衣食父母,是加快发展的最大力量和永恒动力。金杯银杯不如老百姓口碑,金奖银奖不如老百姓夸奖。我们必须保持一心为民的公仆情怀,时刻牢记全心全意为人民服务的根本宗旨,心中装着人民,脑中想着人民,一切为了人民,带着责任和使命干工作,怀着良知和情感创事业。

(八)以建设服务型政府为立足点,推进政府自身建设

加快推进现代新大同建设,必须不断加强政府自身建设。要围绕建设服务型政府,加快政府职能转变,推进政府改革创新,规范政府行政行为,提高政府行政能力,努力建设行为规范、运转协调、公正透明、廉洁高效的政府。

深化行政管理体制改革。进一步理顺部门之间交叉重叠的管理职能,合并相同或相近的职能。一项职能只能由一个部门负责,对可有可无的坚决转移或取消。按照流程最短、效率最高、收费最少、标准最低的要求精简和规范审批事项,再造审批流程,简化审批程序,压缩审批时限。要推行“阳光政务”,凡老百姓自己能够解决的,政府决不干预;凡市场竞争机制能够自行调节的,政府决不干预;凡社会组织能够自我管理的,政府决不干预,切实把政府职能转变为构筑市场载体、培育市场主体、推动社会发展。要以提高办事效率为核心,继续推进效能建设,推进政府行政提速。对该办的事坚决办,不拖;能办的事马上办,不等;难办的事想法办,不推;涉及多个部门的事协调办,不踢。加强便民服务中心建设,优先抓好工商企业项目和房地产开发项目审批服务,做到人进中心、公章进中心、值班领导进中心、所有手续进中心,实行“一窗式受理、一站式办结、一条龙服务”,真正提供快速、高效、便捷的服务。

推进民主政治建设。认真执行人大及其常委会的各项决议、决定,依法向人大及其常委会报告工作,主动接受人大的法律监督和工作监督。自觉接受人民政协的民主监督,虚心听取政协委员的建议和意见。不断提高依法行政的能力,推进法治政府建设,努力做到有权必有责,用权受监督,侵权要赔偿,违法受追究。进一步拓宽与群众沟通协商的渠道,完善市长与人民代表直通车机制。支持人民法院、检察院依法履行职责,支持工会、共青团、妇联等群团组织依法依章工作,参与社会管理和公共服务。进一步加强社会治安综合治理,推进“平安大同”建设。加强民族宗教和侨务工作。努力扩大基层民主,完善民主管理,保障人民权益。

改进工作作风,鼓励干事创业。大力推行“一线工作法”,置身一线,靠前指挥,提高执行力,做到决策在一线落实、问题在一线解决、创新在一线体现、成效在一线检验。要创造干事创业的干部导向,让想干事的有机会、能干事的有舞台、干成事的有地位,让不干事的有压力、干不好事的没市场、干不成事的让位置。要在全市各级政府中树立敢负责、会干事、作风硬、能吃苦的工作作风。我们要为这种作风的干部撑腰壮胆,更要使这样的干部脱颖而出,形成敢抓落实、狠抓落实、会抓落实的生动局面。

坚持勤俭办一切事情的精神。当前我们面临的大事多、难事多、急事多,必须集中财力办大事,集中重点抓突破。劳民伤财、官场做秀的事坚决不干,形式主义、不解决问题的会坚决不开,徒有虚名、没有效果的钱坚决不花。一切经济行为都要讲求经济效益,一切社会行为都要讲求社会效益,一切政务行为都要讲求实际效果。管理就是生产力,要强化“管理立市”的理念,切实加强行政管理,减少和杜绝各种损耗性、流失性的浪费。要加强财政税务审计监督,坚决纠正和打击偷漏税的不法行为,堵塞公共收益流失的漏洞。加强财政预决算管理,减少经常性支出,不断增加民生和公共财力。

切实抓好反腐倡廉建设。清正是为官之德,廉洁是民心所向。“公生明,廉生威”。一个有权威、有效率的政府,一定是干净廉洁的政府。“民不服吾能而服吾公,吏不畏吾严而畏吾廉”。要切实加强反腐倡廉教育,筑牢公务员队伍拒腐防变的思想道德防线。认真落实党风廉政建设责任制,着力建设完善惩治和预防腐败体系。严格落实各项规章制度,凭制度用权、靠制度办事、以制度管人,让权利在监督下实施、在阳光下运行。勤政爱民,简政利民,廉政为民,德政惠民。老老实实做人,干干净净干事,兢兢业业工作,真正做到权为民所用,情为民所系,利为民所谋。

当今时代是一个知识、信息爆炸的时代。一个服务型政府,必然是一个勤于学习、善于学习的政府。我们必须以极大的热忱,学习、学习、再学习,消化和吸收人类文明的一切成果,开阔眼界和胸怀,扩展能力和水平,积累经验和智慧,站在时代的肩膀上,把我们的事情办好,开创大同的新时代。

各位代表,今天的大同,天时、地利、人和,心齐、气顺、劲足,正是我们干实事、干大事、干成事的最佳时机。我们正站在一个新的历史起点上,迈向新的历史征程。历史的眼光注视着我们,人民的期待激励着我们,崇高的事业召唤着我们。让我们紧密团结在以胡锦涛同志为总书记的党中央周围,在省委、省政府和市委的正确领导下,团结一致,求真务实,锐意进取,勤奋工作,创造无愧于时代的新业绩,为开创现代新大同更加美好的明天,实现富民强市的宏伟目标而努力奋斗!
